# Higher Termination of Brachial Artery in Cadavers in the Department of Anatomy of a Medical College: A Descriptive Cross-sectional Study

**DOI:** 10.31729/jnma.6483

**Published:** 2021-08-31

**Authors:** Sanzida Khatun, Diwakar Kumar Shah

**Affiliations:** 1 Department of Anatomy, Nobel Medical College Teaching Hospital, Biratnagar, Nepal

**Keywords:** *arm*, *arteries*, *cadaver*, *forelimb*

## Abstract

**Introduction:**

The main blood supply for arm is provided by the brachial artery. It shows variation in branching and termination patterns in some individuals. Variation in brachial artery may cause difficulties during various clinical and surgical procedures. The present study aims to find out the prevalence of higher termination of brachial artery in cadavers in the department of anatomy of a medical college.

**Methods:**

A descriptive cross-sectional study was carried out from 10th January, 2020 to 20th April, 2021 after the ethical approval was taken from the Institutional Review Committee of Nobel Medical College (reference number: 280/2020). The study was done in 58 upper limbs of 29 properly embalmed cadavers. Convenient sampling was done. They were carefully dissected. The level of termination of brachial artery was noted. The length of the brachial artery and the distance between its termination and the intercondylar line of humerus was recorded.

**Results:**

Higher termination of brachial artery was observed in 3 (5.17%) extremities; one (1.72%) at middle third of arm and two (3.45%) at lower third of arm. In 52 (89.66%) extremities, the site of termination was at the level of neck of radius. The brachial artery terminated a few centimeters below its usual site at the level of upper part of shaft of the radius in 3 (5.17%) extremities.

**Conclusions:**

The prevalence of higher termination of brachial artery in cadavers is slightly lower than the studies performed in similar settings. It is not an uncommon finding. It may have impact on clinical and surgical procedures.

## INTRODUCTION

The main blood supply for arm is provided by the brachial artery. It begins as a continuation of axillary artery at the distal border of teres major and ends by dividing into radial and ulnar arteries at the level of neck of radius. In the arm, it gives branches such as arteria profunda brachii, nutrient artery, superior and inferior ulnar collateral arteries and muscular branches.^1^ It shows variation in branching and termination patterns in some individuals.

Its pulse can be palpated on the anterior aspect of elbow which is widely used to measure the blood pressure, cardiac catheterization for angioplasty and arterial grafting. Variation in brachial artery may cause difficulties during these procedures.^2^ The awareness regarding its variation is required among the clinicians and surgeons before commencing such procedures to avoid complications.

The present study aims to find out the prevalence of higher termination of brachial artery in cadavers.

## METHODS

A descriptive cross-sectional study was carried out in Department of Anatomy of Nobel Medical College Teaching Hospital, Nepal. The ethical approval was taken from the IRC of the institution prior to the study (reference no. IRC-NMCTH 280/2020). The study duration was from 10^th^ January, 2020 to 20^th^ April, 2021. Properly embalmed and formalin fixed cadavers were included in the study. Cadavers with any visible scar or deformity in the superior extremities were excluded from the study.

Sample size was calculated using the formula for estimating population proportion for cross-sectional study,

n = Z^2^ × p × q / e^2^

  = (1.96)^2^ × (0.08) × (1-0.08) / (0.07%)

  = 58

where,

n = minimum sample size required
Z = 1.96 at 95% Confidence Interval (CI)
p = prevalence of higher termination of brachial artery, 8%^3^
q = 1 - p
e = margin of error, 7%

Based on the above formula, the minimum sample size was calculated to be 58. Convenient sampling was done.

Both the upper limbs (n=58) from 29 cadavers were carefully dissected. The brachial arteries were traced proximally to its origin at the lower border of teres major. Distally, it was traced till the upper third of the forearm to evaluate any variation in its terminal branches. The length of the brachial artery was measured in centimeters from lower border of teres major to the level of termination. The distance between intercondylar line of humerus and the the level of termination was recorded proximally for higher terminating brachial arteries and distally for the ones terminating at the level of radius.

The data were entered and analyzed using Statistical Package for the Social Sciences (SPSS) 16.0.

## RESULTS

**Figure 1:**
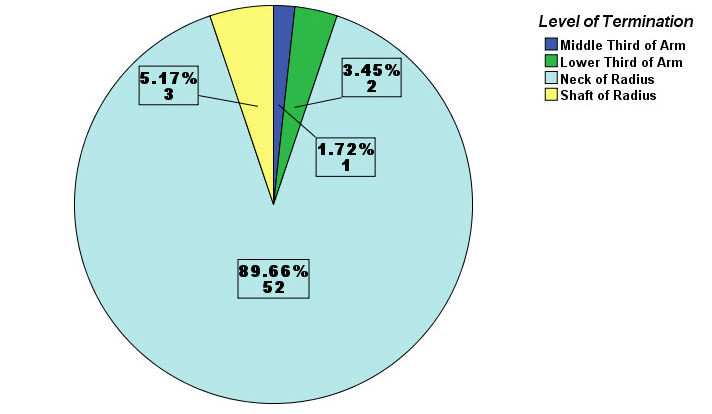
Frequency of different levels of termination of brachial artery.

It was observed that, out of total extremities examined, higher level of termination of brachial artery was found in 3 (5.17%) extremities. Among them, one (1.72%) had terminated at middle third of arm and 2 (3.45%) had terminated at lower third of arm. In 52 (89.66%) extremities, the site of termination was at the level of neck of radius which is the most usual site among majority of the individuals. It was found that in 3 (5.17%) extremities, the brachial artery terminated a few centimeters below its usual site at the level of upper part of shaft of the radius (Figure 1). In this study, there were 19 (65.51%) male cadavers and 10 (34.48%) female cadavers.

All the higher terminations in middle and lower third of the arm were spotted in right arms. Among these 3 extremities, 2 (3.45%) belonged to female cadaver and 1 (1.72%) belonged to male cadaver. Whereas, among the cadavers which had its brachial artery terminating at upper part of shaft of the radius, one cadaver exhibited lower termination bilaterally in both forearms (3.45%). Another cadaver had lower termination of brachial artery in the left forearm (1.72%). Both of them were male cadavers (Table 1).

**Table 1:** Gender-wise frequency of the level of termination of brachial artery.

	Male		Female		Total
	Right (n)	Left (n)	Right (n)	Left (n)	n (%)
Middle Third of Arm	0	0	1	0	1 (1.72)
Lower Third of Arm	1	0	1	0	2 (3.45)
Neck of Radius	17	17	8	10	52 (89.66)
Shaft of Radius	1	2	0	0	3(5.17)

According to the data obtained from the study, the length of the brachial artery ranged from 8.00 cm to 27.50 cm with mean of 23.62 ± 2.98 cm. The distance of level of termination of brachial artery ranged from 1.50 cm to 14.50 cm proximal to the intercondylar line in higher terminating brachial arteries. Among the ones terminating at the level of radius, the distance of level of termination of brachial artery ranged from 1.20 cm to 7.20 cm distal to the intercondylar line (Table 2).

**Table 2:** Length of brachial artery at different levels of terminations.

Length of Brachial artery				
	Mean (cm)	Minimum (cm)	Maximum (cm)	Standard Deviation
Middle Third of Arm	8.00	8.00	8.00	-
Lower Third of Arm	20.40	19.00	21.80	1.98
Neck of Radius	23.83	19.00	27.50	1.94
Shaft of Radius	27.33	27.00	27.50	0.29

## DISCUSSION

Variations of vascular patterns in the upper limb are common findings. They are usually the result of deviation from usual developmental processes during the formation of blood vessels. Variations in the arteries of the upper limb have clinical and surgical significance.^2^ Higher bifurcation of brachial artery is most commonly encountered variation in the upper limb. This study has demonstrated different levels of termination of brachial artery.

In this study, the neck of the radius (89.66%) was the most common level of termination of brachial artery. Three (5.17%) upper limbs had higher termination at middle and lower third of arm and 3 (5.17%) had lower termination at the level of upper part of shaft of radius. In many other studies, the prevalence of higher termination of brachial artery was found to be similar (4%, 6%, 6.5%, 7% respectively) to current study.^4-7^ Slightly varying from current study, other studies showed prevalence of higher bifurcation of brachial artery ranging from 10% to 15%.^8-13^ In contrast to these findings, finding from another author demonstrated greater prevalence (27.6% ) of higher termination of brachial artery.^14^ A study also reveals lower termination of brachial artery at the level of radial tuberosity in 8.6% of upper limbs.^8^

The mean length of brachial artery observed in this study was 23.62 ± 2.98 cm. The mean length values revealed by other studies are higher (27.1 cm and 26.29 cm respectively) than that of the present study.^4,15^ In the current study, the distance of level of termination of brachial artery ranged from 1.50 cm to 14.50 cm proximal to the intercondylar line in higher terminating brachial arteries and 1.20 cm to 7.20 cm distal to the intercondylar line in brachial arteries terminating at the level of radius. A study by Jnanesh, et al. revealed termination level ranged from 1cm to 19cm proximal to intercondylar line in higher bifurcations.^11^ A study by

Kaur A reports termination level ranged from 7cm to 20 cm proximal to intercondylar line in higher bifurcations.^8^ In a case report in India, bilateral higher bifurcations of brachial artery was reported. The right brachial artery termination was 7.5 cm proximal to intercondylar line and the left was 10.5 cm proximal to intercondylar line.^16^ Another case report revealed unilateral higher termination of brachial artery at the junction of upper and middle one-third of the left arm.^17^

Although this study wasn't aimed to evaluate other kind of variations found in upper limb vasculature, there are many other variations reported by various studies. Other variations related to brachial artery reported are duplications, superficial brachial artery, accessory brachial artery, dilated tortuous brachial artery and trifurcations.^2,18^ These variations may have occurred due to multiple plexiform sources, crossed haemodynamic predominance between primitive deep and superficial arterial segments and regression of arterial segments that have minor haemodynamic significance.^2^ Such unexpected variations during routine clinical and surgical procedure may cause hazards. The superficial radial artery may be mistaken for vein causing reflex vascular occlusion by accidental cannulation resulting in risk of gangrene.^18^ A case was reported where brachial artery was explored for acute arterial insufficiency of right upper limb. Residual embolus at high bifurcating point of brachial artery was detected and thrombectomy of high originating ulnar artery was done.^19^

The brachial and radial arteries are also widely used for measuring blood pressure, arterial grafting, cardiac catheterization for angioplasty and preoperative mapping for AV access in advanced and end stage renal disease.^13,18^ All of these procedures may be affected by unrecognized variations in these arteries. Some studies have revealed inferior secondary and functional patency rates, frequent anastomotic lesions and non-maturation of brachio-cephalic arterio-venous fistula in patients with higher termination of brachial artery.^14,20^

The sample size taken in this study was quite less owing to the minimal availability of the cadavers which limits the scope of the study. Additional research with larger sample size will be required to evaluate further accurate prevalence of variations in vasculature and its relation with the outcomes of the clinical and surgical procedures.

## CONCLUSIONS

The prevalence of higher termination of brachial artery in cadavers is slightly lower than the studies performed in similar settings. It is not an uncommon finding. It may have impact on clinical and surgical procedures. Thus, pre-procedural angiographic and Doppler ultrasound imagings are of considerable importance before commencement of any relevant procedures.
